# Different Effects of Quercetin Glycosides and Quercetin on Kidney Mitochondrial Function—Uncoupling, Cytochrome C Reducing and Antioxidant Activity

**DOI:** 10.3390/molecules27196377

**Published:** 2022-09-27

**Authors:** Kristina Zymone, Raimondas Benetis, Darius Trumbeckas, Ingrida Baseviciene, Sonata Trumbeckaite

**Affiliations:** 1 Laboratory of Biopharmaceutical Research, Institute of Pharmaceutical Technologies, Lithuanian University of Health Sciences, Sukileliu Av. 13, LT-50162 Kaunas, Lithuania; 2 Department of Drug Chemistry, Faculty of Pharmacy, Lithuanian University of Health Sciences, Sukileliu Av. 13, LT-50162 Kaunas, Lithuania; 3 Department of Urology, Medical Academy, Lithuanian University of Health Sciences, Eivenių G. 2, LT-50009 Kaunas, Lithuania; 4 Department of Obstetrics and Gynaecology, Lithuanian University of Health Sciences, Eiveniu Str. 2, LT-50009 Kaunas, Lithuania; 5 Neuroscience Institute, Lithuanian University of Health Sciences, Sukileliu Av. 13, LT-50162 Kaunas, Lithuania; 6 Department of Pharmacognosy, Faculty of Pharmacy, Lithuanian University of Health Sciences, Sukileliu Av. 13, LT-50162 Kaunas, Lithuania

**Keywords:** quercetin, rutin, hyperoside, isoquercitrin, kidney mitochondria, respiration rates, uncoupling, antioxidant, cytochrome c reducing properties

## Abstract

Flavonols are found in plants as aglycones and as glycosides. Antioxidant activity of flavonols may occur via several mechanisms within the cell, and mitochondria as a target may play an important role. There is a lack of information about the influence of the sugar moiety on biological activity of flavonoid glycosides. The aims of study were to investigate the effects of quercetin and its glycosides on mitochondrial respiration rates at various metabolic states, and to evaluate their antioxidant potential using chemical and biological approaches. Mitochondrial function was measured using an oxygraphic method, cytochrome c reduction spectrophotometrically, H_2_O_2_ generation in mitochondria fluorimetrically, and antioxidant activity of flavonoids using an HPLC-post column system. Our data revealed that quercetin and its glycosides isoquercitrin, rutin, and hyperoside uncouple kidney mitochondrial respiration (increasing the State 2 respiration rate) and significantly reduce cytochrome c. Moreover, quercetin, and its glycosides decrease the production of mitochondrial H_2_O_2_ and possess radical scavenging and ferric reducing capacities. The highest activity was characteristic for quercetin, showing that the sugar moiety significantly diminishes its activity. In conclusion, our results show the efficient radical scavenging, ferric and cytochrome c reducing capacities, and uncoupling properties of quercetin and its glycosides, as well as the importance of the sugar residue and its structure in the regulation of kidney mitochondrial function.

## 1. Introduction

Flavonols are the most widespread group of flavonoids in plants [1]. Quercetin is one of the most abundant flavonoids in the human diet and is one of the most frequent flavonoids in fruits, vegetables, and beverages [2].

In vitro and in vivo studies have revealed that quercetin possesses a wide spectrum of biological effects that include anti-inflammatory [3,4], antilipoperoxidant [5], antitumoral [6,7], antimicrobial [8], anti-diabetic [9,10], anti-atherosclerotic [11], and cardioprotective [12,13] activities. It is well known that the biological activity of flavonoids is mostly related to their antioxidant potential [14]. According to antioxidant activity, various groups of flavonoids can be arranged in the following order: flavan-3-ols > flavonols > chalcones > flavones > flavanones > isoflavones [15]. However, it has been reported that flavonol quercetin is one of the strongest free radical scavengers in vitro [16], being even more potent than some of flavan-3-ols [15].

The intracellular antioxidant activity of flavonoids may occur via several mechanisms within various subcellular compartments, and mitochondria may play an important role, since they are an important site of superoxide anion production and very sensitive to oxidative stress. In a physiological environment, free radicals are formed during metabolic processes, and they participate in energy production, cell growth regulation, transmission of intercellular signals. If the balance between free radical production and defense mechanisms is disturbed, free radicals may react with lipids, proteins, and DNA, thereby causing structural changes in the structure of proteins and damaging membranes and DNA. Reactive oxygen species (ROS) have been implicated in the pathogenesis of inflammation, aging, and carcinogenesis [17].

Mitochondria constitute one of the most important ROS producers in the cells and may be one of the targets of oxidative stress. On the other hand, they are important not only for the generation of ROS, but also for the suppression of ROS production via the mechanism of slight uncoupling of oxidative phosphorylation. Moreover, mitochondria produce ATP and play a pivotal role in cellular metabolism and survival. Cytochrome c is an important component of the mitochondrial electron transport chain and exists in oxidized (Fe^3+^) and reduced (Fe^2+^) forms. Research suggests that the redox state of cytochrome c plays an important role in the regulation of the intrinsic pathway of apoptosis [18], because the oxidized form of cytochrome c can induce caspase activation while the reduced form does not. Therefore, the targeted regulation of mitochondrial function by biologically active compounds is of great importance. Polyvalent biological activity of flavonoids is mostly related to their aglycone structure. However, flavonoids usually do not occur in plants as aglycones. To accumulate flavonoids in vacuoles it is necessary to increase their polarity, therefore flavonoids frequently occur as glycosides in plants. The sugar moiety can determine molecular properties that are important for the biological activity of particular compounds.

Our previous studies on heart mitochondria showed that some flavonoids possess mitochondrial uncoupling activity, in a concentration-dependent and structure-dependent manner [19]. We revealed that quercetin has the highest effect at the lowest concentration used, whereas quercitrin had a clearly lower effect. Slight uncoupling of mitochondria might be useful, because it leads to a decrease in membrane potential and prevents the generation of excessive ROS in mitochondria. Strategies for reducing or preventing the generation of ROS, and the regulation of the redox state of cytochrome c, are very important, especially for ischemia/reperfusion injury in vital organs such as the heart or kidney. In the kidney, ischemia/reperfusion injury (during partial nephrectomy and a surgical procedure) may contribute to disturbances of postoperative kidney function and high mortality rates; therefore, the search for protective compounds against oxidative stress-induced damage is important. Data on the targeted regulation of kidney mitochondrial function by quercetin and its glycosides are very limited, therefore the aims of our study were (1) to investigate the effects of quercetin and its glycosides on mitochondrial respiration rates at various metabolic states, and (2) to evaluate their antioxidant potential assessed by a chemical approach (post-column free radical scavenging and ferric reducing antioxidant power approach), and a biological approach including cytochrome c reducing properties and H_2_O_2_ production in rat kidney mitochondria.

## 2. Results

### 2.1. Effects of Quercetin and Its Glycosides on Mitochondrial Respiration Rates

**Figure 1:**
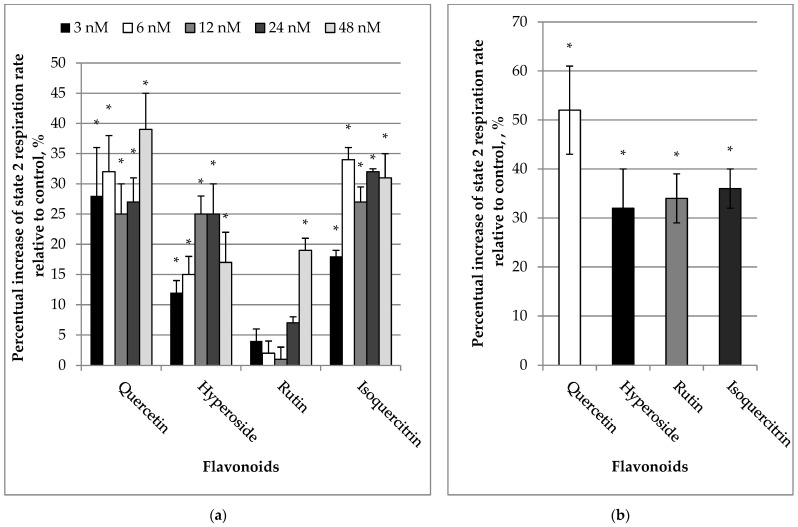
(**a**) Effect of flavonoids (3–48 nM) on State 2 respiration rates in kidney mitochondria. (**b**) Effect of flavonoids (10 µM) on State 2 respiration rate in kidney mitochondria. The mitochondrial State 2 respiration (V_2_) rate was recorded in medium supplemented with mitochondria and substrates (5 mM glutamate + 5 mM malate). The respiration rate of mitochondria without added flavonoids was considered as a control. The effect was calculated according to Formula (1). * Statistically significant difference (*p* < 0.05) compared to control (State 2 respiration rate without flavonoids).

We investigated the effects of quercetin and its glycosides (hyperoside, rutin, isoquer-citrin) on kidney mitochondrial functions with glutamate/malate as substrates. Our results revealed that quercetin starting from a concentration of 3 nM caused a marked increase (by 28%, *p* < 0.05) in mitochondrial State 2 (non-phosphorylating) respiration rate (Figure 1a). Isoquercitrin and hyperoside had lower effects (18% and 12%, respectively). Oxygen consumption by mitochondria at State 2 increased in a concentration-dependent manner with increasing flavonoid concentrations. Thus, at a concentration of 6 nM, the stimulating effects of quercetin, isoquercitrin, and hyperoside were 32, 34 and 15% respectively, whereas rutin was found to be ineffective. Higher concentrations (12–48 nM) of quercetin, isoquercitrin, and hyperoside caused statistically significant increases in State 2 respiration rate by 25–39, 23–31, and 17–25%, respectively. Rutin had no effect within the 3–24 nM range, and only stimulated the State 2 respiration rate by 19% (*p* < 0.05) starting at 48 nM. Higher concentrations of flavonoids also demonstrated the uncoupling effect, and increased proton leak. Therefore, when concentrations of quercetin were increased up to 10 µM, the stimulatory effect on State 2 respiration rate reached 52 ± 9% (*p* < 0.05, Figure 1b). Hyperoside, rutin, and isoquercitrin also induced significant stimulation of mitochondrial respiration by 32 ± 8, 34 ± 5, and 36 ± 4%, respectively (*p* < 0.05).

**Figure 2:**
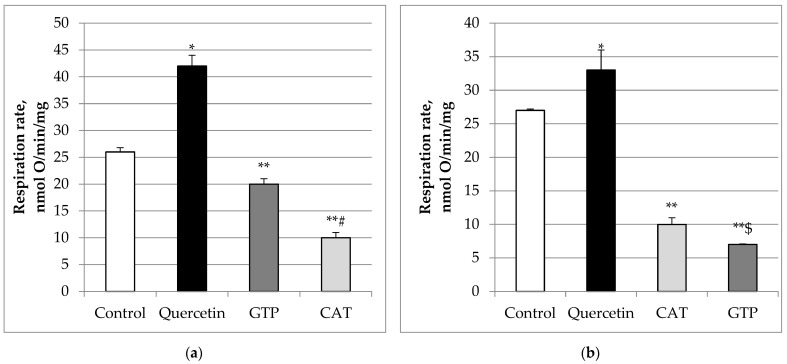
Effect of GTP (**a**) and CAT (**b**) on mitochondrial State 2 respiration rate stimulated by quercetin. The mitochondrial State 2 respiration (V_2_) rate was recorded in a medium supplemented with mitochondria and substrates (5 mM glutamate + 5 mM malate) in the presence of quercetin (9 µM). Then, guanosine triphosphate (GTP, 30 μM), an inhibitor of UCP, was added followed by the addition of carboxyatractyloside (CAT, 4 μM), an inhibitor of the adenine nucleotide translocator (Figure 2a). Similar experiments were performed in a different order (instead of CAT, GTP was added at first, and then CAT (Figure 2b). Mitochondrial respiration rates are expressed as nmolO/min/mg. * Statistically significant difference (*p* < 0.05) compared to control (i.e., to State 2 respiration rate without quercetin). ** Statistically significant difference (*p* < 0.05) compared to State 2 respiration rate (V2) in the presence of quercetin. **# Statistically significant difference (*p* < 0.05) as compared to GTP-group. **$ Statistically significant difference (*p* < 0.05) compared to CAT-group.

A significant part of proton leak is dependent on the adenine nucleotide trans-locator (ANT), the most abundant carrier protein in the mitochondrial inner membrane, and to a smaller extent on uncoupling protein (UCP). Therefore, we tested which of these pathways may be activated by quercetin. Carboxyatractyloside (CAT), an inhibitor of the adenine nucleotide translocator, statistically significantly (*p* < 0.05) reduced (by 76%) quercetin-stimulated State 2 respiration rate (Figure 2b). Further addition of guanosine triphosphate (GTP, an inhibitor of UCP) diminished the CAT-inhibited respiration by 83%, *p* < 0.05, thereby having an additive effect. We also revealed that the addition of GTP, reduced quercetin-stimulated State 2 respiration rate (Figure 2a). by 52%, and addition of CAT suppressed it further (by 76%). There was a statistically significant difference between these groups (*p* < 0.05). Thus, our data show that UCP may be involved in the mechanism of quercetin induced uncoupling as well as ANT.

**Figure 3:**
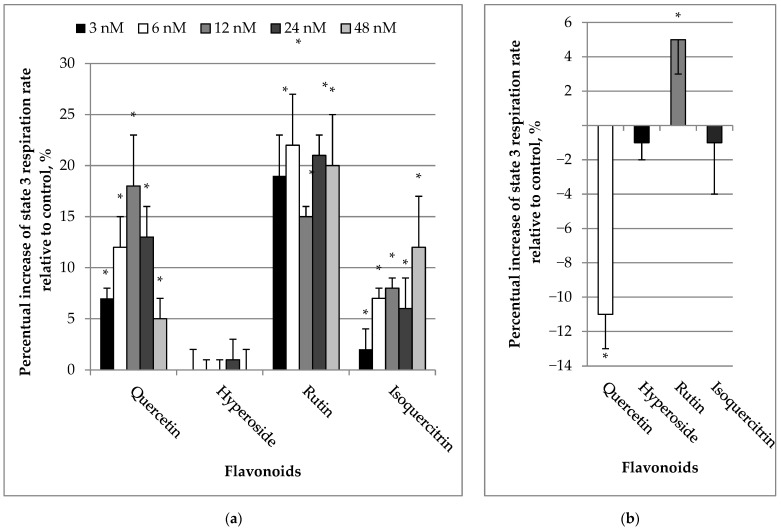
(**a**) Effect of flavonoids (3–48 nM) on State 3 respiration rates in kidney mitochondria. (**b**) Effect of flavonoids (10 µM) on State 3 respiration rate in kidney mitochondria. The mitochondrial Sstate 3 respiration rate was recorded in a medium supplemented with mitochondria, substrates (5 mM glutamate + 5 mM malate and 1 mM ADP) as described in Materials and Methods. The respiration rate of mitochondria without added flavonoids was considered as the control. The effect was calculated according to the Formula (2). * Statistically significant difference (*p* < 0.05) compared to control (State 3 respiration rate without flavonoids).

We also measured the effects of quercetin and its glycosides on the ADP-stimulated (State 3) respiration rate of mitochondria. Quercetin even at low concentrations (3–48 nM) significantly increased State 3 respiration rate by 7–18% (*p* < 0.05) (Figure 3a). Similar trends were observed for rutin and isoquercitrin (stimulation by 15–22%, 2–12%, respectively; *p* < 0.05). Hyperoside had no effect at all. The highest concentration of quercetin (10 µM) induced a decrease in State 3 respiration rate by 11 ± 2% (*p* < 0.05), whereas hyperoside and isoquercitrin at the same concentration did not present any significant effect (Figure 3b). On the contrary, rutin increased the State 3 respiration rate by 5% (*p* < 0.05).

### 2.2. Effects of Quercetin and Its Glycosides on Reduction of Cytochrome C

**Figure 4:**
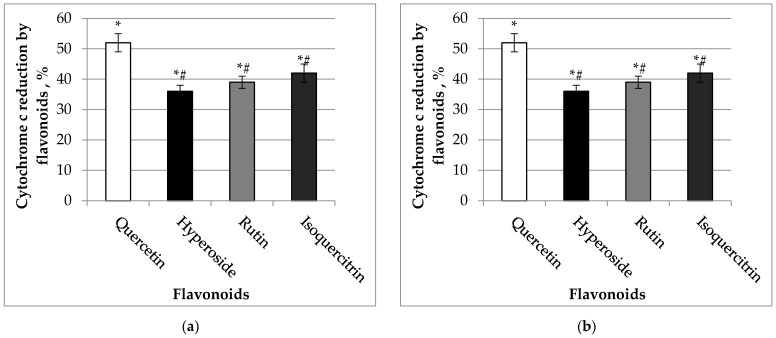
(**a**) Effect of 10 µM of flavonoids on cytochrome c reduction. (**b**) Effect of 20 µM of flavonoids on cytochrome c reduction. For the cytochrome c reduction, the spectra of flavonoids were recorded over 500–600 nm and taken as the experimental blanks and then 32 M cytochrome c was added, and the cytochrome c spectrum was recorded every 3 min as described in Material and Methods. The height of absorption peak at 550 nm was taken and compared to the absorption peak of completely reduced cytochrome c (after addition of dithionite) at the end of the test. The effect was calculated according to Formula (3). * Statistically significant difference (*p* < 0.05) compared to control (without flavonoids). *# Statistically significant difference (*p* < 0.05) compared to the quercetin group.

The capacity of flavonols to reduce cytochrome c, a component of the mitochondrial electron transport chain, is shown in Figure 4. Our data revealed that quercetin was the most potent, and this aglycone induced cytochrome c reduction by 52 and 62%, *p* < 0.05, at concentrations of 10 µM and 20 µM, respectively. The other flavonoids also significantly reduced cytochrome c at the same concentrations.

### 2.3. Effects of Quercetin and Its Glycosides on Mitochondrial Production of H_2_O_2_

**Figure 5:**
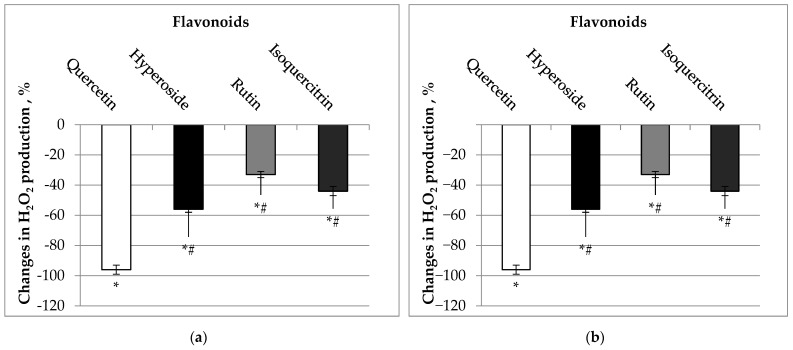
(**a**) Effect of 10 µM of flavonoids on mitochondrial production of H_2_O_2_. (**b**) Effect of 30 µM of flavonoids on mitochondrial production of H_2_O_2_. Mitochondrial production of H_2_O_2_ was measured as described in Material and Methods. * Statistically significant difference (*p* < 0.05) compared to control (without flavonoids). The effect was calculated according to Formula (4). *# Statistically significant difference (*p* < 0.05) compared to quercetin-group.

We also tested whether quercetin and its glycosides affect mitochondrial H_2_O_2_ production. Our data revealed that quercetin at concentrations of 10–30 µM decreased H_2_O_2_ production almost completely by 96 and 97%, isoquercitrin by 44 and 60%, hyperoside by 56 and 66%, and rutin by 33 and 53% (Figure 5), *p* < 0.05. The observed potent ability of quercetin and its glycosides to counteract H_2_O_2_ production suggests that investigated flavonoids may affect the electron flow through the mitochondrial electron transport chain.

### 2.4. Radical Scavenging and Ferric Reducing Capacities of Quercetin and Its Glycosides

**Figure 6:**
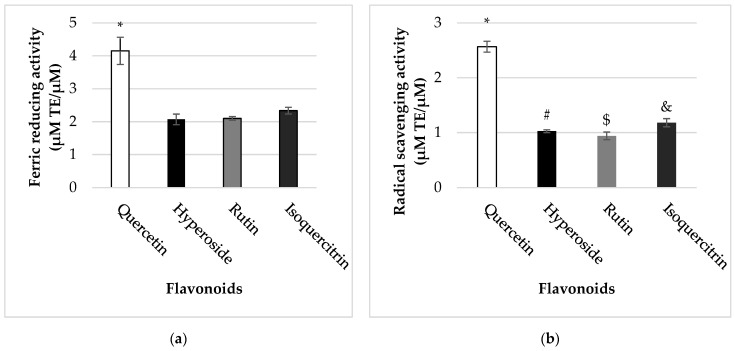
Ferric reducing (**a**) and radical scavenging (**b**) properties of flavonoids. Ferric reducing and radical scavenging activities of flavonoids were measured as described in Material and Methods. The antioxidant activity of flavonoids is expressed as µM Trolox equivalents (TE) for 1 µM of certain flavonoid. * Statistically significant difference (*p* < 0.05) compared to hyperoside, rutin, and isoquercetin. # Statistically significant difference (*p* < 0.05) compared to quercetin. $ Statistically significant difference (*p* < 0.05) compared to quercetin and isoquercetin. & Statistically significant difference (*p* < 0.05) compared to quercetin and rutin.

The antioxidant potential of quercetin and its glycosides was evaluated using ABTS^•+^ and FRAP post-column assays. This coupled approach enabled us to investigate the different modes of antioxidant activity of flavonoids (free radical scavenging and reducing abilities, respectively). Our data revealed that quercetin possessed stronger (*p* < 0.05) antioxidant capacity in both in vitro systems (2.57 ± 0.10 µM TE/µM of quercetin in ABTS^•+^ and 4.15 ± 0.41 µM TE/µM of quercetin in FRAP post-column assays) than its glycosidic forms (Figure 6). Among them, quercetin with a glucose moiety attached to the C-3 (isoquercitrin) was found to be more active in both assays. However, there were no statistically significant differences between ferric reducing capacities of quercetin glycosides (*p* > 0.05).

## 3. Discussion

The findings of this study are that quercetin and its glycosides isoquercitrin, rutin, and hyperoside (1) uncouple kidney mitochondrial respiration (increase the State 2 respiration rate), in a dose-dependent manner; (2) possess antioxidant activity (radical scavenging and reducing capacities *in vitro*) and suppress the mitochondrial H_2_O_2_ production, and (3) rapidly and directly reduce cytochrome c. Among investigated flavonoids, the mostly active was quercetin followed by its glycosides.

In this study the effects of quercetin and its glycosides were tested at the level of kidney mitochondria. Mitochondria, the main producers of ATP, essential for the cell functioning, are very sensitive to oxidative stress, which may occur after infarction, during partial nephrectomy and surgical procedures. Therefore, the investigation of mitochondria-targeted compounds, which possess antioxidant activity and might mitochondria-protective effects, are of great importance.

To check the effects of quercetin and its glycosides on kidney mitochondrial respiration rates at various metabolic states we used a wide spectrum of their concentrations. The concentrations of flavonoids were used based on our previous experiments with heart mitochondria [19], since we had revealed previously that flavonoids affect heart mitochondria at nM concentrations, whereas in liver mitochondria higher amounts of flavonoids are needed [20].

Our earlier findings with heart mitochondria also revealed the uncoupling properties of flavonoids in the nM range (State 2 respiration rate was stimulated by 100–110% at a very low (3.6 nM) quercetin concentration [19]. In order to achieve the similar effect, much higher (by nine-fold) concentrations of hyperoside (32.7 nM) and by 20-fold concentrations of rutin (72.7 nM) were used. Thus, our present findings suggest that kidney mitochondria are less sensitive to the uncoupling effect of flavonoids; at similar concentrations the uncoupling activity was two- to three-fold less than in heart mitochondria. This is not surprising, as Dorta et al. [20,21] did not find any stimulating effect in rat liver mitochondria respiring on succinate or glutamate + malate, even at more than a thousand times higher concentration of quercetin (at 25 μM and 50 μM). These differences might be caused by different levels of UCP and ANT in kidney, heart, and liver mitochondria, or by variations in composition of mitochondrial membranes. By investigating mechanism of action, we revealed that, ANT and UCP are involved in uncoupling of kidney mitochondria by quercetin, as both, CAT (an inhibitor of ANT) and GTP (an inhibitor of UCP) suppressed quercetin-stimulated State 2 respiration rate. Similar findings are described in our earlier studies with heart mitochondria [22]. Our present study revealed that quercetin in the nM range, and some of its glycosides (rutin and isoquercitrin), induced the stimulation of State 3 respiration rate, while at µM concentrations quercetin had a negative effect. Hyperoside and isoquercitrin at the same µM concentrations had no effect. Thus, moderate mitochondrial uncoupling (when State 3 respiration rate is not inhibited) might be beneficial for the cell as it could suppress ROS generation by mitochondria, which has been confirmed by our results. Quercetin (aglycone) among all investigated flavonoids (i.e., quercetin with sugar residues) was the most potent. Moreover, we revealed that quercetin and its glycosides are powerful cytochrome c reductants. This fact is important, as reduced cytochrome may block apoptosis. An earlier study of Lagoa et al. [23] revealed that the flavonoids epicatechin, quercetin, and kaempferol exhibit cytochrome c reducing activities. Taken together, these facts show that mitochondria may be an important target for querctin and other flavonoids. As several studies have already shown that reduced cytochrome c is less potent in activation of caspase than cytochrome c in its oxidized form, biologically active compounds that reduce cytochrome c could suppress caspase activation by the reduction of cytosolic cytochrome c. We assume that quercetin and its glycosides might be useful therapeutic agents in oxidative-stress-related diseases (such as kidney ischemia/reperfusion injury occurring during partial nephrectomy) due to cytochrome c reducing and antioxidant properties [24,25,26].

We revealed that the effects on mitochondria depend not only on the dose but also on the structure of flavonoids, and the sugar residue plays an important role. For example, the mostly active mitochondrial uncoupler is aglycone quercetin followed by isoquercitrin (quercetin-3-O-β-d-glucopyranoside) and hyperoside (quercetin-3-O-galactoside). Rutin (quercetin-3-O-rutinoside) has the lowest uncoupling activity among all investigated flavonoids in this study. Thus, glycosylation clearly diminishes the uncoupling properties of flavonoids. Cytochromes c reducing activities are listed in a similar manner: quercetin > isoquercitrin > rutin > hyperoside. Radical scavenging and ferric reducing capacities are also listed in a similar way: quercetin > isoquercitrin > rutin ≥ hyperoside.

Generally, the ability of organic compounds to efficiently scavenge free radicals is attributed to the generation of more stable radicals, which, in turn, depends on electron delocalization capacity of these molecules. Thereby the planarity of some structures here plays an important role. In this study, quercetin was found to be the most potent in both chemical and biological in vitro antioxidant assays. Our data revealed that antioxidant (antiradical and FRAP reducing) capacity of quercetin is stronger compared to its glycosides. Similar effects were obtained by measuring cytochrome c reducing capacity. This is consistent with earlier findings, since quercetin possesses the main structural features required for effective antioxidant activity, including the o-dihydroxy (catechol) arrangement in the B-ring, increasing the stability of oxidized flavonoid radicals through H-bonding or electron-delocalization; the 2,3-double bond in conjugation with a 4-carbonyl group in the C-ring is capable of delocalizing π-electrons and thereby stabilizing resulting radicals after H-abstraction, and three free hydroxyl groups (at C-3, C-5 and C-7) acting as scavengers of free radicals [24]. Additionally, quercetin is a planar molecule in which electron delocalization between the B-ring and C-ring is favored.

The results of this study show that quercetin and the studied glycosides (hyperoside, rutin, isoquercitrin), with unaltered catecholic moiety in the B-ring, possess free radical scavenging ability in both non-organelle (scavenging of ABTS radical cation) and biological systems (suppression of H_2_O_2_ production in isolated mitochondria). Furthermore, increasing evidence indicates that glycosylation of flavonoids substantially reduces their antioxidative capacity [24]. Our results also demonstrate that quercetin possesses greater radical scavenging ability than its derivatives (hyperoside, rutin, and isoquercitrin) in both ABTS^•+^ post-column and in mitochondrial H_2_O_2_ generation assays. This is in agreement with other scientific studies, showing that since free phenolic hydroxyl groups are necessary for hydrogen abstraction and radical scavenging, the substitution of these functionalities with glycosidic moieties alters the hydrophilic and lipophilic balance of the flavonoid structure and, most likely, negatively influences antioxidant properties [24]. Moreover, the position of the sugar moiety plays an important role. All studied glycosides have a key structural feature required for effective radical scavenging, namely an enol moiety in the C-ring [25]. O-glycosylation at position C-3 interferes with the coplanarity of the B-ring with the rest of the flavonoid, thereby conjugation and delocalization of electrons could not be ensured in the molecules of the glycosides [26]. As a result, O-glycosylation of flavonoid aglycones decreases their antioxidant potential and effects on mitochondria.

## 4. Materials and Methods

### 4.1. Chemicals

Glutamic acid, succinic acid, adenosine-5′-diphosphate sodium salt (ADP), cytochrome c from bovine heart, malic acid, ethylene glycol-bis-(b-aminoethylether)-N,N,N′N′-tetra acetic acid (EGTA), KH_2_PO_4_, TrisHCl, ethylenediamine tetra-acetic acid (EDTA), amytal, carboxyatractyloside (CAT), guanosine triphosphate (GTP)quercetin, acetonitrile, 2,2-azinobis (ethyl-2,3-dihydrobenzothiazoline-6-sulphonic acid) diammonium salt (ABTS), potassium persulfate, iron(III) chloride hexahydrate (FeCl_3_·6H_2_O), 2,4,6-tripyridyl-s-triazine (TPTZ), hydrochloric acid, 6-hydroxy-2,5,7,8-tetramethylchroman-2-carboxylic acid (Trolox) were obtained from “Sigma–Aldrich GmbH”, Steinheim, Germany. Sucrose, mannitol, HEPES, KCl, MgCl_2_, trifluoroacetic acid, hyperoside, isoquercitrin, rutin were obtained from “Carl Roth Gmbh”, Karlsruhe, Germany.

### 4.2. Preparation of Isolated Kidney Mitochondria

Preparation of kidney mitochondria of Wistar rats weighing 200–250 g was performed using a differential centrifugation method as described before [27]. The experimental procedures were performed with permission from the Lithuanian Committee of Good Laboratory Animal Use Practice (No. 0228/2012).

### 4.3. Measurement of Mitochondrial Respiration

Mitochondrial respiration (oxygen consumption) was measured at 37 °C as described before [27]. Mitochondrial State 2 (non-phosphorylating) respiration (*V_2_*) rate was recorded in medium supplemented with mitochondria (0.5 mg/mL) and substrates (5 mM glutamate + 5 mM malate). Then, 1 mM ADP was added, and State 3 respiration rate was measured. Finally, the ADP/ATP translocator inhibitor carboxyatractyloside (CAT) 4 μM was added and State 4 respiration rate was measured. To check the effects of flavonoids on State 2 respiration rate, first we registered mitochondrial State 2 respiration rate (for 1 min) and then the different concentrations of respective flavonoids were added. The effect (changes in State 2 respiration rate (in %)) was calculated according to the formula:

(1) $$Effect\;\left(\%\right)=\left(\frac{V_{2F}}{V_{2}}\times 100\right)-100,$$

*V*_2*F*_—mitochondrial State 2 respiration rate with flavonoids.

*V*_2_—mitochondrial State 2 respiration rate without flavonoids.

Note: State 2 respiration rate in control group, i.e., without flavonoids was equated to 100 percent.

To check the effects of flavonoids on State 3 respiration rate, in the medium supplemented with mitochondria, and substrates (5 mM glutamate + 5 mM malate), and 1 mM ADP, State 3 respiration rate was measured for 1 min and then the different concentrations of respective flavonoids were added. The effect (changes in State 3 respiration rate (in %)) was calculated according to the formula:

(2) $$Effect\;\left(\%\right)=\left(\frac{V_{3F}}{V_{3}}\times 100\right)-100,$$

*V*_3*F*_—mitochondrial State 3 respiration rate with flavonoids.

*V*_3_—mitochondrial State 3 respiration rate without flavonoids.

Note: State 3 respiration rate in control group, i.e., without flavonoids was equated to 100 percent.

To check the mechanism of action of flavonoids on State 2 respiration rate, mitochondrial State 2 respiration (*V_2_*) rate was recorded in the medium supplemented with mitochondria and substrates (5 mM glutamate + 5 mM malate) in the presence of quercetin, registered for 1 min, and then the guanosine triphosphate (GTP, 30 μM), an inhibitor of UCP, was added. After 1 min registration, carboxyatractyloside (CAT, 4 μM), an inhibitor of adenine nucleotide translocator, was added. Similar experiments were performed in a different order (instead of CAT GTP was added at first), and only then CAT.

### 4.4. Measurement of Cytochrome C Reduction Level

The reduction of cytochrome c was recorded spectrophotometrically as described before [28]. The effect (cytochrome c reduction by flavonoids (in %)) was calculated according to the formula:

(3) $$Effect\;\left(\%\right)=\frac{cytochrome\;c\;reduction\;level\;with\;flavonoids\times 100}{dithionite\;reduced\;cytochrome\;c\;level},$$

Note: Dithionite-reduced cytochrome c level was taken as 100%.

### 4.5. Measurement of H_2_O_2_ Generation

H_2_O_2_ generation was estimated fluorimetrically at 37 °C using an Amplex Red detection system with a Thermo Scientific Fluoroscan Ascent plate reader, as described before [22] with 5 mM glutamate + 5 mM malate as substrates in the medium for kidney mitochondrial respiration [27]. The effect (changes in H_2_O_2_ production (in %)) was calculated according to the formula:

(4) $$Effect\;\left(\%\right)=\left(\frac{H_{2}\;O_{2}\;production\;with\;flavonoids\times 100}{H_{2}\;O_{2}\;production\;without\;flavonoids}\right)-100,$$

### 4.6. On-Line Measurement of Flavonoids Antioxidant Activity Using HPLC-Post Column System

Analysis of antioxidant activity was performed using a Waters 2695 Alliance system (Waters, Milford, MA, USA) with a photodiode array detector Waters 996. Separation was performed using an ACE column (C18, 150 mm × 4.6 mm, particle size 3 μm) with a 3 mm ACE guard column (C18, 20 mm × 4.0 mm) (Aberdeen, Scotland) thermostated at 25 °C. The mobile phase consisted of 0.05% (*v*/*v*) trifluoroacetic acid solution in water (solvent A) and acetonitrile (solvent B). Solutions of flavonoids (0.002 M) were analyzed under isocratic conditions (50% solvent B). Eluent flow rate was 0.5 mL min-1, and the injection volume 1 μL. ABTS^•+^ and FRAP solutions were prepared and HPLC-post column conditions were applied as described by Raudonis et al. [29]. The ferric reducing and radical scavenging activities of flavonoids were assessed by standard antioxidant Trolox. Calibration curves of Trolox were made. To generate the calibration curve, ethanolic solutions of Trolox were injected into the HPLC post-column system and analyzed under the isocratic conditions mentioned above. The antioxidant activity of flavonoids was expressed as µM Trolox equivalents (TE) for 1 µM of certain flavonoid.

### 4.7. Statistical Data Processing Methods

Data were evaluated using statistical methods. Descriptive statistical indicators, Student’s *t* test, ANOVA method were used. *p* < 0.05 was taken as the level of significance. Normality and homogeneity of variance of the data were confirmed using a Shapiro-Wilk test and Levene’s test, respectively. Results are expressed as mean ± standard deviation (SD) of three replicates. Calculations were made using computer programs Microsoft Office Excel 2003 and SPSS 20.0 software (Chicago, IL, USA).

## 5. Conclusions

In conclusion, our results show that (1) quercetin and its glycosides (hyperoside, rutin, and isoquercitrin) uncoupled mitochondria in a dose-dependent and sugar residue-dependent manner and possess radical scavenging, ferric and cytochrome c reducing capacities; (2) quercetin was the most active among investigated flavonoids, and (3) glycosylation of quercetin diminished all investigated effects, namely mitochondrial uncoupling, the radical scavenging, ferric and cytochrome c reducing capacities. The observed dose-dependent uncoupling effect of quercetin involves ANT-mediated and UCP-mediated proton conductance of the inner mitochondrial membrane and may have protective mechanisms against oxidative stress induced by excessive ROS production. Thus, due to radical scavenging, ferric and cytochrome c reducing capacities, and uncoupling properties, quercetin and its glycosides may offer potential for the design of mitochondria-targeted compounds against oxidative stress.

## Data Availability

Not applicable.

## References

[1] Xiao J., Kai G., Yamamoto K., Chen X. (2013). Advance in Dietary Polyphenols as α-Glucosidases Inhibitors: A Review on Structure-Activity Relationship Aspect. Crit. Rev. Food Sci. Nutr..

[2] Lamson D.W., Brignall M.S. (2000). Antioxidants and Cancer, Part 3: Quercetin. Altern. Med. Rev..

[3] Hämäläinen M., Nieminen R., Vuorela P., Heinonen M., Moilanen E. (2007). Anti-Inflammatory Effects of Flavonoids: Genistein, Kaempferol, Quercetin, and Daidzein Inhibit STAT-1 and NF-ΚB Activations, Whereas Flavone, Isorhamnetin, Naringenin, and Pelargonidin Inhibit Only NF-ΚB Activation along with Their Inhibitory Effect on INOS Expression and NO Production in Activated Macrophages. Mediat. Inflamm.

[4] García-Mediavilla V., Crespo I., Collado P.S., Esteller A., Sánchez-Campos S., Tuñón M.J., González-Gallego J. (2007). The Anti-Inflammatory Flavones Quercetin and Kaempferol Cause Inhibition of Inducible Nitric Oxide Synthase, Cyclooxygenase-2 and Reactive C-Protein, and down-Regulation of the Nuclear Factor KappaB Pathway in Chang Liver Cells. Eur. J. Pharmacol..

[5] Oyewopo A.O., Adeleke O., Johnson O., Akingbade A., Olaniyi K.S., Areola E.D., Tokunbo O. (2021). Regulatory Effects of Quercetin on Testicular Histopathology Induced by Cyanide in Wistar Rats. Heliyon.

[6] Zhang H., Zhang M., Yu L., Zhao Y., He N., Yang X. (2012). Antitumor Activities of Quercetin and Quercetin-5′,8-Disulfonate in Human Colon and Breast Cancer Cell Lines. Food Chem. Toxicol..

[7] Shan B.E., Wang M.X., Li R.Q. (2009). Quercetin Inhibit Human SW480 Colon Cancer Growth in Association with Inhibition of Cyclin D1 and Survivin Expression through Wnt/β-Catenin Signaling Pathway. Cancer Investig..

[8] Hirai I., Okuno M., Katsuma R., Arita N., Tachibana M., Yamamoto Y. (2010). Characterisation of Anti-Staphylococcus Aureus Activity of Quercetin. Int J Food Sci Technol.

[9] You Q., Chen F., Wang X., Jiang Y., Lin S. (2012). Anti-Diabetic Activities of Phenolic Compounds in Muscadine against Alpha-Glucosidase and Pancreatic Lipase. LWT-Food Sci. Technol..

[10] Jeong S.M., Kang M.J., Choi H.N., Kim J.H., Kim J.I. (2012). Quercetin Ameliorates Hyperglycemia and Dyslipidemia and Improves Antioxidant Status in Type 2 Diabetic Db/Db Mice. Nutr. Res. Pract..

[11] Kleemann R., Verschuren L., Morrison M., Zadelaar S., van Erk M.J., Wielinga P.Y., Kooistra T. (2011). Anti-Inflammatory, Anti-Proliferative and Anti-Atherosclerotic Effects of Quercetin in Human in Vitro and in Vivo Models. Atherosclerosis.

[12] Annapurna A., Reddy C.S., Akondi R.B., Rao S.R.C. (2009). Cardioprotective Actions of Two Bioflavonoids, Quercetin and Rutin, in Experimental Myocardial Infarction in Both Normal and Streptozotocin-Induced Type I Diabetic Rats. J. Pharm. Pharmacol..

[13] Chen Y.W., Chou H.C., Lin S.T., Chen Y.H., Chang Y.J., Chen L., Chan H.L. (2013). Cardioprotective Effects of Quercetin in Cardiomyocyte under Ischemia/Reperfusion Injury. Evidence-based Complement. Altern. Med..

[14] Hernández-Rodríguez P., Baquero L.P., Larrota H.R. (2019). Flavonoids: Potential Therapeutic Agents by Their Antioxidant Capacity. Bioactive Compounds: Health Benefits and Potential Applications.

[15] Cai Y.Z., Sun M., Xing J., Luo Q., Corke H. (2006). Structure-Radical Scavenging Activity Relationships of Phenolic Compounds from Traditional Chinese Medicinal Plants. Life Sci..

[16] Russo M., Spagnuolo C., Tedesco I., Bilotto S., Russo G.L. (2012). The Flavonoid Quercetin in Disease Prevention and Therapy: Facts and Fancies. Biochem. Pharmacol..

[17] Chow J.M., Shen S.C., Huan S.K., Lin H.Y., Chen Y.C. (2005). Quercetin, but Not Rutin and Quercitrin, Prevention of H_2_O_2_-Induced Apoptosis via Anti-Oxidant Activity and Heme Oxygenase 1 Gene Expression in Macrophages. Biochem. Pharmacol..

[18] Brown G.C., Borutaite V. (2008). Regulation of Apoptosis by the Redox State of Cytochrome c. Biochim. Biophys. Acta-Bioenerg..

[19] Trumbeckaite S., Bernatoniene J., Majiene D., Jakštas V., Savickas A., Toleikis A. (2006). The Effect of Flavonoids on Rat Heart Mitochondrial Function. Biomed. Pharmacother..

[20] Dorta D.J., Pigoso A.A., Mingatto F.E., Rodrigues T., Prado I.M.R., Helena A.F.C., Uyemura S.A., Santos A.C., Curti C. (2005). The Interaction of Flavonoids with Mitochondria: Effects on Energetic Processes. Chem. Biol. Interact..

[21] Dorta D.J., Pigoso A.A., Mingatto F.E., Rodrigues T., Pestana C.R., Uyemura S.A., Santos A.C., Curti C. (2008). Antioxidant Activity of Flavonoids in Isolated Mitochondria. Phytother. Res..

[22] Baliutyte G., Baniene R., Trumbeckaite S., Borutaite V., Toleikis A. (2010). Effects of Ginkgo Biloba Extract on Heart and Liver Mitochondrial Functions: Mechanism(s) of Action. J. Bioenerg. Biomembr..

[23] Lagoa R., Graziani I., Lopez-Sanchez C., Garcia-Martinez V., Gutierrez-Merino C. (2011). Complex I and Cytochrome c Are Molecular Targets of Flavonoids That Inhibit Hydrogen Peroxide Production by Mitochondria. Biochim Biophys Acta Bioenerg.

[24] Heim K.E., Tagliaferro A.R., Bobilya D.J. (2002). Flavonoid Antioxidants: Chemistry, Metabolism and Structure-Activity Relationships. J. Nutr. Biochem..

[25] Amić A., Lučić B., Stepanić V., Marković Z., Marković S., Dimitrić Marković J.M., Amić D. (2017). Free Radical Scavenging Potency of Quercetin Catecholic Colonic Metabolites: Thermodynamics of 2H^+^/2e^−^ Processes. Food Chem..

[26] Soobrattee M.A., Neergheen V.S., Luximon-Ramma A., Aruoma O.I., Bahorun T. (2005). Phenolics as Potential Antioxidant Therapeutic Agents: Mechanism and Actions. Mutat. Res.-Fundam. Mol. Mech. Mutagen..

[27] Baniene R., Trumbeckas D., Kincius M., Pauziene N., Raudone L., Jievaltas M., Trumbeckaite S. (2016). Short Ischemia Induces Rat Kidney Mitochondria Dysfunction. J. Bioenerg. Biomembr..

[28] Skemiene K., Rakauskaite G., Trumbeckaite S., Liobikas J., Brown G.C., Borutaite V. (2013). Anthocyanins Block Ischemia-Induced Apoptosis in the Perfused Heart and Support Mitochondrial Respiration Potentially by Reducing Cytosolic Cytochrome c. Int. J. Biochem. Cell Biol..

[29] Raudonis R., Raudone L., Jakstas V., Janulis V. (2012). Comparative Evaluation of Post-Column Free Radical Scavenging and Ferric Reducing Antioxidant Power Assays for Screening of Antioxidants in Strawberries. J. Chromatogr. A.

